# Counter hegemony, popular education, and resistances: A systematic literature review on the squatters’ movement

**DOI:** 10.3389/fpsyg.2022.1030379

**Published:** 2022-10-20

**Authors:** Julia Ballesteros-Quilez, Pablo Rivera-Vargas, Judith Jacovkis

**Affiliations:** ^1^Faculty of Psychology, University of Barcelona, Barcelona, Spain; ^2^Department of Teaching and Learning and Educational Organization, University of Barcelona, Barcelona, Spain; ^3^Facultad de Educación y Ciencias Sociales, Universidad Andrés Bello, Santiago, Chile

**Keywords:** squatting movement, squatters, anti-capitalism, social movements, neoliberalism, self-management, insurgency, social transformation

## Abstract

The squatting movement is a social movement that seeks to use unoccupied land or temporarily or permanently abandoned buildings as farmland, housing, meeting places, or centers for social and cultural purposes. Its main motivation is to denounce and at the same time respond to the economic difficulties that activists believe exist to realize the right to housing. Much of what we know about this movement comes from the informational and journalistic literature generated by actors that are close or even belong to the movement. However, there is also a significant diversity of knowledge and scientific evidence on the squatters’ movement that is being produced by academia and that is worth knowing and grouping together. With the aim of defining and understanding how the squatters’ movement is constituted and organized, and how it acts, this research analyzes what the scientific literature affirms about it. Through qualitative research based on the systematic literature review (SLR) method, information was sought in the Web of Science (WOS) and Scopus databases. The initial universe of 262 articles was finally reduced to a sample of 32 articles. These have been analyzed by means of a categorized classification content analysis. The results obtained allow us to establish the state of the art on the squatting movement, placing special emphasis on its dynamics of resistance, its process of political subjectivation and its mechanisms of action and self-management. The study suggests that the movement is understood based on collective actions with a political role of resistance to neoliberalism and the inequalities it generates, and of response to the basic and social needs of the communities through self-management.

**Systematic review registration:**

[doi: 10.5281/zenodo.7179670], identifier [7179670].

## Introduction

The development of the squatting movement, born in the 1960s in Western Europe, has been a clear response to urban policies, especially those linked to the housing market, and has proposed an alternative way of constructing individual and collective life. Thus, it has opted for a politically conscious, participatory, self-managed, and creative life option, without dependence on established hierarchies and outside the dynamics of the world of consumption and the market. The emergence of this movement, as well as other new social movements, can be interpreted from the theory of Inglehart (2018) who investigates the process of transformation from a “materialist society” to a “post-materialist society.” In materialistic societies, values were conditioned by material needs. In contrast, post-materialist values have driven claims to issues related to the preservation of autonomy, political participation, identity, or quality of life.

Occupation as a phenomenon refers to the action of squatting, to the very fact of illegally entering and inhabiting someone else’s property, whether to use it as a home, to house political projects or for both objectives (Anton, 2020). Despite their diversity and the richness of their demands, it is common to restrict, reduce and simplify the scope of the social movement to the act of opening and entering a building (Anton, 2020). For Pruijit (2013), it is in this way that an specific and disruptive action becomes a symbol of the occupation, which overshadows its other dimensions. However, the process of squatting, as well as the different ways of inhabiting occupied spaces, make it a complex and diverse movement that has evolved and changed over time.

For Martínez (2011) and Morawski (2019), the expansion of the squatters’ movement since the 1970s is due to transnational imitation and activists’ personal connections, which constitute social and political networks. In the same vein, for Anton (2020), its survival over time has been possible thanks to internal discussions and debates that reoriented some of its priorities and tools of struggle. Indeed, currently some squatting social centers are not only providers of leisure but also of services such as legal advice, food collection or the promotion of self-occupation (Rivero and Abasolo, 2010; Morawski, 2019). Thanks to their social and anti-hegemonic aims, links are established with other social, political, or neighborhood movements, so that squatting is adopted by the daily and political struggle of many collectives that initially did not see it as a valid and efficient response or action to achieve their objectives (Cattaneo and Martínez, 2014; Anton, 2020). In fact, the movement, which for many years was marginalized due to the questioning and attack on private property it entails, has seen its legitimacy increase over the last decade for these linkages and interrelationships (Anton, 2020; Nowicki, 2020).

The squatting movement proposes an alternative path to the construction of individual and collective life (Staniewicz, 2011). In this vein, for Martínez (2019) many of its participants promote collective direct action, self-management and communal lifestyles that challenge capitalist urbanization, housing speculation and unsustainable and alienated lives. Squatting communities provide identity resources and enable the development of commonly shared skills that are transferable to other territories (Bouillon, 2009).

Although the contexts and projects are considerably differentiable, authors such as Martínez (2011, 2016), Cattaneo and Martínez (2014), and Morawski (2019) argue that there are also common patterns in the squatting movement. In the case of Europe, for example, some important motivations for all types of occupation have been: the need for social housing for vulnerable groups; the social and cultural resignification of unsatisfied and unused urban and rural spaces; the search for identity and well-being in urban territorial contexts inhabited by the working class; the search for empowerment and greater neighborhood organization; and, in general, anti-hegemonic resistance to housing policies subjugated to the market and financial speculation, and to the promotion of the gentrification of historic neighborhoods.

In relation to its definition, the literature so far offers numerous divergent interpretations that convey the difficulty of defining the squatters’ movement (Pruijit, 2013). At the same time, none of these interpretations can be considered incorrect, since the squatters’ movement presents a great variability and diversity of projects, even depending on the national or local context in which they are developed (Pruijit, 2013; Morawski, 2019).

As a social and political movement, the occupation has been approached by literature from many perspectives, reflecting its own complexity and heterogeneity: historical, political, anthropological, and sociological (Alonso, 2015). At the same time, there is not only academic literature, but also many sources of counter-information and alternatives to the *mass-media* generated by the squatters’ movement itself. These sources are easy to access, but they are clearly discursive vehicles as their main objectives are to promote citizen support, make alliances, seek recognition as cultural centers and disseminate the movement’s ideas through social networks (Venegas Ahumada, 2014). Therefore, we cannot consider that there is no bias in the information they offer.

Given the complexity of the movement on a social level, and the existence of numerous bibliographical precedents that address its different dimensions, in this article we are particularly interested in learning about the various focuses of interest of scientific research on the phenomenon of the squatter movement. Without wishing to delegitimize the importance of the more informative and political references in the field, we are interested above all in answering the following questions based on the available scientific evidence:

What is the squatter movement? What are its main dynamics of counter-hegemonic action?, What are its main focuses of interest?, What are its main mechanisms of action and self-management?, And what social resistances does it encounter?

With the aim of answering these questions, a systematic literature review (SLR) of scientific articles on the squatter movement published between 2019 and 2021 has been developed. The present work, in this sense, aims to establish what is the state of the art in the scientific literature and what are its main focuses.

## Approach to the theoretical framework. An approach to its history and main characteristics

The squatter movement is considered by some authors as one of the New Social Movements (NMSs) that emerged in the late 1960s (Pruijit, 2013; Subirats, 2013). Unlike the classical movements, they have a networked structure, are more informal and unstable and follow an organizational model more enthusiastic (Calhoun, 1993 cited by Pruijit, 2013) and detached from the relations of production that characterized traditional movements (Chihu, 1999). In any case, like traditional movements, NMS are forms of collective action that respond to the abuses of economic and political powers and involve processes of consciousness-raising for social emancipation (Vargas, 2008), thus contributing to the generation of identities and new ways of living (Chihu, 1999).

The “squatting” phenomenon, as we understand it today, responds to the need for accommodation and the need for spaces that serve as base of operations for alternative political and cultural activities. The occupation of empty houses and buildings to satisfy these needs has its origins in Britain in the 1960s and early 1970s, when countercultural groups settled more or less permanently in dwellings that were not used by their owners (Cattaneo and Martínez, 2014). The movement was very strong due to the large number of abandoned dwellings and the fact that most of them were owned by local councils, which lacked funds to modernize them and therefore left them unused.

The movement quickly spread to Denmark, the Netherlands and Germany, with different nuances in each case. In the late 1960s, German cities such as Berlin, Hamburg, and Freiburg began to be subject to numerous “hausbesetzung” (squatting in Deutsche). The first wave of Germany squatting was linked to the student revolution of 1968. The second wave began in the years 1978–1979 with the declaration of “redevelopment” zones in old Berlin districts; the situation became scandalous: while countless flats were left empty, the demand for housing soared. In these circumstances, the squatting movement reappeared, and its first targets were precisely the houses affected by these redevelopment plans. In the Netherlands, in the late 1970s and early 1980s, students and former provost occupied uninhabited buildings proposed for demolition in the wake of the ideas put forward by the provos and kabouters. The occupation (“Krakers” in Dutch) was very popular among Amsterdam’s youth. Over the years, the Netherlands has become the European country where the squatting movement has stabilized the most, as it has found an attitude of dialogue and support from administrations (Kriesi, 1989; Morawski, 2019; Van der Steen et al., 2020). In this sense, the enforcement of the Law 12305 established that a property could only be left unused for 1 year, and the municipality of Amsterdam, for example, publishes a catalog of occupiable houses when the owners, in addition to having them empty, do not comply with minimum conservation requirements.

Depending on the motivations for squatting a space, building, or dwelling, Pruijit (2013) presents a classification along five dimensions:

1. Deprived occupation includes people who, because of their poverty, do not have access to any kind of housing. For them, the only alternative to occupation is homelessness. In this case, the main demand is not structural but seeks to meet an individual need.
2. Occupancy as an alternative housing strategy is not as restrictive as the previous one, as it does not necessarily imply conditions of poverty. In this case, squatting is seen as an alternative to renting.
3. Occupation as entrepreneurship is that which allows any project to be developed without the bureaucracy involved in doing so in other ways. This includes neighborhood centers, squatters’ bars, or personal or collective social actions and projects.
4. Conservationist occupation is one that aims to conserve and preserve the urban landscape, avoiding urbanization and renewal, or slowing down gentrification processes.
5. The occupation as a political action sustains an anti-system positioning and identifies itself as revolutionary with autonomous ideas.

In synthesis, we can say that the squatting movement, despite its great heterogeneity, shares in its majority of expressions a motivation of transformation or resistance in an emancipatory key or, at the very least, a practice that is subversive insofar as it represents a transgression of the right to private property. To squat, in this sense, is indeed to violate private property, but not with a lucrative interest but with an intention that goes from survival to social transformation (Squatting Europe Kollective [SQEK], 2010).

Beyond Pruijit (2013) classification, to which we will return later, the squatting movement has been a key actor in bringing to the table the tensions raised by the exploitation of cities by markets (Polanska and Weldon, 2020). In this sense, their actions have not been limited to the occupation of spaces in passive terms, but rather to their use as places of collective construction and resistance to hegemony. Thus, various researchers have been interested in movement as an element of social transformation in different contexts. Staniewicz (2011) points out that the squatting movement is an object of study for both urban sociology and the sociology of social movements.

On the one hand, from a point of view of the study of urban dynamics, Guzmán (2008, cited by Staniewicz, 2011) argues that squatting is an adaptive instrument in the face of the lack of housing characteristic of many European cities, and that it plays an active role in the reform and improvement of urban ecology. In this sense, the squatting movement seeks to reclaim the Right to the City. As described by Lefebvre (1967 cited by Molano, 2016 p. 4), this right is the right of all urban dwellers to build, decide and create the city, making it a privileged space for anti-capitalist struggle. Thus, Lefebvre proposed it as an alternative to the social and urban depoliticization promoted by modern states (Molano, 2016). However, despite the transformative and radical potential of the Right to the City, institutions and administrations have used it discursively but have also detached it from its initial political and ideological content, which has given rise to weak participatory processes and forms of self-management and eventually has contributed to sustain and give more importance to certain municipal participatory processes (Mayer, 2012; Dee, 2018).

On the other hand, approaches from the sociology of social movements highlight some key aspects of the squatters’ movement as its diversity and radicalism, especially in comparison with other forms of participation and existing urban movements. This is manifested in the promotion of building takeovers and the development of strategies of everyday grassroots and neighborhood self-management of urban spaces. Martínez (2011), in fact, proposes to speak of “squatter movements” in the plural, due to their heterogeneity and variability depending on the local and historical contexts in which they have been developing. These contexts, nevertheless, share characteristics typical of the neoliberal model of the city such as inequalities, social polarization, and the precariousness of living conditions (Llobet, 2004).

Despite these common factors, the squatting movement cannot be considered solely as a reactive movement to the system, but as a generator of alternatives that materialize in particular experiences of self-management, construction of daily coexistence, reflection on the contradictions and interpersonal and collective conflicts that arise, etc. (Llobet, 2004). In this sense, squatted social centers and squats constitute political experiences of contestation to political and urban transformations in neoliberal contexts (Miró, 2008).

Both for its role in the configuration of urban scenarios and for its characteristics as a social movement, the squatters’ movement is a counter-hegemonic movement insofar as, through its practice, it publicly and collectively questions what is defined as normal, taken for granted, and that forms part of common sense: private property, individualism, or the need for institutions to organize collective practices due to the impossibility of self-management. On the other hand, the movement represents an experience of popular education. Beyond its different expressions, the movement’s practices involve collective learning and knowledge-building processes that have had a transformative impact in many of the contexts in which they have developed, both at the neighborhood and city level, and in terms of the activists’ experiences (Rivera-Vargas et al., 2022). Through the creation of open social centers, participation in neighborhood assemblies and other initiatives linked to their environments, the squatting movement has contributed to generating reflection and critical thinking among its activists, who have been formed in these environments as political subjects.

This brief review of the emergence and evolution of the squatting movement and its framing as a counter-hegemonic social movement capable of promoting popular education strategies in its spaces of intervention leads us to ask how the scientific literature has delved into some of its characteristics. The methodological approach presented in the following section, which has guided this analysis, aims to find out how research on this movement has responded to the questions formulated in the introduction.

## Materials and methods

The method used to carry out this study is documentary analysis, a procedure based on the need to facilitate individuals’ access to information sources, bearing in mind that the volume of information production has been increasing (Peña and Pirella, 2007).

In order to determine the state of the art on the squatter movement, as well as to analyze, identify and synthesize the scientific information available in this field in order to make it more accessible and comprehensible, a SLR has been carried out (Sanz, 2020; Fardella et al., 2022). This SLR is based on the PRISMA 2020 protocol (Moher et al., 2009; Page et al., 2021; Sosa-Díaz et al., 2022).

Systematic literature review is a type of scientific research whose main purpose is to objectively and systematically integrate the results of previous studies on the same research problem, thus determining the state of the art in the chosen field of study (Sánchez-Meca and Botella, 2010).

Based on the article’s guiding questions, this study used systematic and explicit methods to locate, select and critically appraise relevant research (Sánchez-Meca and Botella, 2010), so that valid and objective conclusions could be drawn about the questions posed.

### Sources of information

For the collection of information, search strategies were applied in different databases, identifying studies by date (last 3 years) and type of document (journal articles). The search was limited by language to articles in English, Spanish, Catalan, and French. This study selection process was carried out independently by two reviewers acting in different phases. Specifically, these reviewers divided the document searches by databases (WOS and Scopus).

### Search strategies

The search included different combinations of the words *squatting OR squatters* together with other keywords: *urban squatting, social centers, social work, neighborhood, political squatting, neoliberalism, social transformation, social change*, and *community*, using the Boolean operator “AND,” and specifying that the words appear in the title or between the keywords.

Due to the polysemy of the word “*squatting*,” in order to obtain results on our research problem, the search was limited to the following research domains in Web of Science (WOS): *Anthropology, Cultural studies, Political science, Psychology, Geography, Psychology applied, Psychology experimental, History, regional urban planning, History of social sciences, Cultural studies, Demography, International relations, Social issues, Development studies, Law, social sciences interdisciplinary, Sociology, Economics, Education educational research.*

In Scopus the search was also limited to the following subject areas: *Social sciences, Arts and humanities, Business, Management and accounting, Economics, econometrics and finance, Psychology, Environmental Science, Earth and Planetary Sciences, Multidisciplinary* (see Table 1).

**TABLE 1:** Database search description.

Database	Description
Sequence of filters in SCOPUS	TITLE-ABS-KEY TITLE-ABS-KEY + PUBYEAR > 2018 + DOCTYPE (AR) TITLE-ABS-KEY + PUBYEAR > 2018 + DOCTYPE (AR) Subject area: *Social sciences, Arts and humanities, Business, Management and accounting, Economics, econometrics and finance, Psychology, Environmental Science, Earth and Planetary Sciences, Multidisciplinary*.
Sequence of filters in WOS	TITLE-ABS-KEY TITLE-ABS-KEY + PUBYEAR > 2018 + DOCTYPE (AR) TITLE-ABS-KEY + PUBYEAR > 2018 + DOCTYPE (AR) Research domains: *Anthropology, Cultural studies, Political science, Psychology, Geography, Psychology applied, Psychology experimental, History, regional urban planning, History of social sciences, Cultural studies, Demography, International relations, Social issues, Development studies, Law, social sciences interdisciplinary, Sociology, Economics, Education educational research*

### Selection process

**FIGURE 1:**
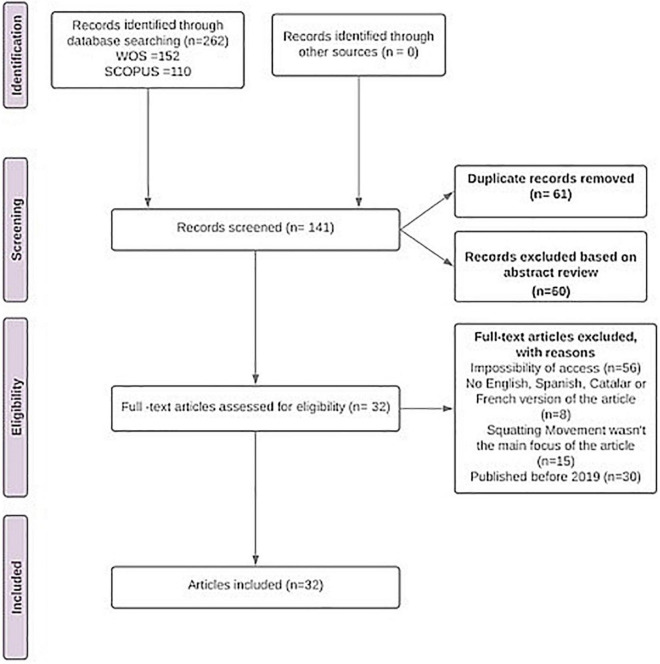
Flowchart based on the PRISMA 2020 statement (Page et al., 2021). Source: own elaboration.

The Prisma protocol suggests the execution of four phases in the SLR. These are: Identification, Screening, Eligibility, and Inclusion (see Figure 1). In these phases, the criteria for selection and elimination of texts were grouped as follows:

In the identification phase, once the different word combinations had been included in the WOS and Scopus databases, a total of 262 articles were found. In the screening phase, duplicate articles were eliminated (*n* = 61) and also those which, based on the reading of the abstract, were not related to the object of study or the guiding question (*n* = 60), resulting in a total of 141 articles.

In the eligibility phase, after reading or attempting to read the articles, a total of 109 of the 141 resulting from the screening process were eliminated. This elimination was carried out on the basis of four criteria: because of impossibility of access or paid access (*n* = 56), because they were not available in Catalan, Spanish, or English (*n* = 8), because their main focus was not the squatter movement (*n* = 15) and, finally, because they were published before 2019 (*n* = 30). No exclusion criteria were established based on the territorial area studied by the articles, as the aim was to obtain a global vision of the squatters’ movement.

Thus, the final sample in this SLR included 32 articles published between 2019 and 2021 (Table 2).

**TABLE 2:** Articles included in the SLR.

NO.	References	Publication	Title
1	Aceros et al., 2019	Journal of youth studies	“Often it is because of who is doing it.” The production of a youth subculture’s image through talk.
2	Asara, 2019	Partecipazione e conflitto.	“The redefinition and co-production of public services by Urban movements: The Can Batllo social innovation in Barcelona.”
3	Caciagli, 2019	Antipode	Housing Squats as “Educational Sites of Resistance”: The Process of Movement Social Base Formation in the Struggle for the House.
4	Campbell, 2019	Anthropology today	Of squatting amid capitalism on Yangon’s industrial periphery.
5	Dadusc, 2019b	Citizenship studies	The Micropolitics of border struggles: migrants’ squats and inhabitance as alternatives to citizenship.
6	Dadusc, 2019a	City	Enclosing autonomy: The politics of tolerance and criminalization of the Amsterdam squatting movement
7	Dadusc et al., 2019	Citizenship studies	Introduction: citizenship as inhabitance? Migrant housing squats versus institutional accommodation
8	De Biasi, 2019	City	Squatting and adverse possession: Countering neighborhood blight and disinvestment
9	Lauri, 2019	Subjectivity	Social movements, squatting and communality: ethical practices and re-subjectification processes
10	Maestri, 2019	International journal of urban and regional research	The Nomad, The Squatter and the State: Roma Racialization and Spatial Politics in Italy.
11	Martínez, 2019	Culture unbound	Good and bad squatters? Challenging hegemonic narratives and advancing anti-capitalist views of squatting in western European cities
12	Raimondi, 2019	Citizenship studies	For “common struggles of migrants and locals.” Migrant activism and squatting in Athens
13	Starecheski, 2019	American ethnologist	Social movements, squatting and communality: ethical practices and re-subjectification processes
14	Atabien and Tekdemir, 2020	Studies in psychology – psikoloji calismalari dergisi	Identity Positionings in Squatters’ Framings of Don Quijote Social Centre
15	Burgum, 2020	Journal of urban history	This City Is An Archive: Squatting History and Urban Authority
16	Chiodelli et al., 2020	Progress in planning	The production of informal space: A critical atlas of housing informalities in Italy between public institutions and political strategies
17	De Moor, 2020	International journal of urban and regional research	Alternatives to Resistance? Comparing Depoliticization in Two British Environmental Movement Scenes
18	Dos Santos, 2020	Partecipazione e conflitto	Squatting, commons and conflict: A discussion of squatting’s challenges to the commons.
19	González et al., 2020	Participation and conflict	Squatted and self-managed social Centers in Mexico City: Four case studies from 1978–2020.
20	Ighe, 2020	Participation and conflict	Empty space, open space. Claiming, reaching and remembering common ground in ubran squats. Haga in the 1980s.
21	Malik et al., 2020	Journal of housing and the built environment	Investigation of informal housing challenges and issues: experiences from slum and squatter of Lahore.
22	Martínez, 2020a	Partecipazione e conflitto	Urban commons from an anti-capitalist approach.
23	Martínez, 2020b	Routledge handbook of contemporary European social movements: protest in turbulent times	European squatters’ movements and the right to the city.
24	Novák, 2020	Social movement studies	Every city needs a klinika: the struggle for autonomy in the post-political city.
25	Novák and Kuřík, 2020	Journal of urban affairs	Rethinking radical activism: Heterogeneity and dynamics of political Squatting in Prague after 1989.
26	Nowicki, 2020	Environment and planning c-politics and space	Is anyone home? Appropriating and re-narrativising the post-criminalization squatting scene in England and Wales.
27	Polanska and Weldon, 2020	Participation and conflict	In Search of Urban Commons Through Squatting: The Role of Knowledge Sharing in the Creation and Organization of Everyday Utopian Spaces in Sweden.
28	Soresina, 2020	Journal of urban history	The Housing Struggle in Milan in the 1970s: Influences and Particularities.
29	Van der Steen et al., 2020	Journal of urban history	Who Are the Squatters? Challenging Stereotypes through a Case Study of Squatting in the Dutch City of Leiden, 1970–1980.
30	Yardımcı, 2020	Antipode	State Stigmatization in Urban Turkey: Managing the “Insurgent” Squatter Dwellers in Dikmen Valley.
31	Zaman, 2020	Transactions of the institute of British geographers	Neighborliness, conviviality, and the sacred in Athens’ refugee squats
32	Karaliotas and Kapsali, 2021	Antipode	Equals in Solidarity: Orfanotrofio’s Housing Squat as a Site for Political Subjectification Across Differences Amid the “Greek Crisis.”

The 32 selected texts were subjected to a content analysis based on open coding (Strauss and Corbin, 2002). In addition, five axial categories were established in which the different codes identified were grouped and related: (1) Origin and conceptualization of the squatter movement. (2) Counter-hegemonic action against Neoliberalism and Capitalism. (3) Links with the community. (4) Squatter movement and migration. (5) Limitations and resistances to the squatting movement (see Table 3). The documents that make up the sample were analyzed according to these axial categories in a spreadsheet (Sosa-Díaz et al., 2022).

**TABLE 3:** Categorization and number of articles that mention each category.

NO.	Categories about the squatting movement	Articles
1	Origin and conceptualization of the squatter movement.	25/32
2	Resistance to neoliberalism and capitalism.	26/32
3	Squatters’ movement and migration.	13/32
4	Community outreach.	25/32
5	Limitations and resistances.	25/32

## Results

The results obtained through the SLR are grouped into the five axial categories mentioned above.

### Origin and conceptualization of the squatter movement

There are numerous definitions of what the squatting movement is and what its main actions are in the literature analyzed. For numerous authors, occupation as a phenomenon refers to the action of squatting, to the very fact of entering someone else’s property illegally and inhabiting it, whether to use it as a home, to house political projects or for both objectives (Cattaneo and Martínez, 2014 cited by González et al., 2020; Aceros et al., 2019; De Biasi, 2019; Martínez, 2019; Atabien and Tekdemir, 2020; Chiodelli et al., 2020). In normative terms, occupation would consist of the action of occupying a property without the prior consent of the owner and, above all, without a legal right to that property (Campbell, 2019; Atabien and Tekdemir, 2020; Chiodelli et al., 2020).

The squatting movement historically emerges as a collective response to housing crises (Pruijit, 2013 cited by Atabien and Tekdemir, 2020; Campbell, 2019; Nowicki, 2020). Therefore, according to the literature analyzed, it is not surprising that there is a strong link between this movement and political activism as an alternative and counter to capitalism (Squatting Europe Kollective [SQEK], 2010 cited in Polanska and Weldon, 2020; Nowicki, 2020).

Several authors frame the squatters’ movement as an example of the politics of the act, which are contrary to the politics of demand. The politics of the act are based on the premise that freedom and emancipation should not be asked for, but should be built and lived, creating alternatives to the state and social organization (Dadusc, 2019a; Ighe, 2020; Novák and Kuřík, 2020).

Occupation is a phenomenon that continues to occur in Europe and affects the global scale, but according to the literature analyzed, it is difficult to compare situations in different development contexts (Raimondi, 2019; Martínez, 2020a; Soresina, 2020). It is not, therefore, a homogeneous movement. On the contrary, it is extremely heterogeneous, and takes different forms depending on the country, city and even areas and administrations within the same city (Gelder, 2013 cited in Dadusc, 2019a; Malik et al., 2020; Martínez, 2020a). However, and in spite of the difficulties in generalizing, for various authors, the communication relations, the activists’ travels, and the mutual influence in the anti-capitalist resistance practices mean that the movement can be considered transnational (Owens et al., 2013 cited in Martínez, 2020a).

Most of the self-managed squatting projects coincide in the fact of meeting social needs (Caciagli, 2019; Dadusc et al., 2019; González et al., 2020) through direct action (Dos Santos, 2020). Firstly, by meeting the need for housing (Caciagli, 2019; Karaliotas and Kapsali, 2021) but also other needs such as the creation of common goods and spaces that enable socialization and the generation of cohesive communities (Dos Santos, 2020; Polanska and Weldon, 2020).

#### Identities and types of occupation: Who squats?

The image of the squatter as a political militant who generates continuous confrontations with the police and violent conflicts emerged during the 1980s in Europe (Van der Steen et al., 2020). However, to adhere to the myth of the militant squatter often depicted as a white, thin, young man excludes all other people who are also part of the movement: “apolitical” squatters, migrants, women, etc. (Kadir, n.d. cited by Van der Steen et al., 2020).

There are examples such as the Haga neighborhood in Götemborg, with a large squatter focus, where activists claimed to have built a tolerant community composed of all kinds of people: children with dysfunctional families, alcoholics, as well as other people suffering from social exclusion, such as transgender people (Ighe, 2020). Informants also stressed the importance of relationships, links and learning between young squatters and older people who had been or were squatters (Ighe, 2020; Soresina, 2020), thus breaking the established stereotype.

The classification and characterization of the squatting movement is difficult. According to Martínez (2020a), the most relevant work in the field is the approach of Pruijit (2013) who presents, as we have mentioned in the theoretical framework, a classification of urban occupation according to the motivations of such occupation. This classification has been widely applied and criticized, as some of these configurations can be interrelated (Martínez, 2020a). Furthermore, some authors argue that the practice of occupation, as long as it is collective, is inherently political as it subverts one of the basic principles of the hegemonic order: private property (Caciagli, 2019; Polanska and Weldon, 2020).

### Counter-hegemonic action toward neoliberalism and capitalism

The neoliberal era has seen changes in urban spaces, accelerated in many cities by the growth of urbanization- and restructuring-oriented capitalism (Polanska and Weldon, 2020; Soresina, 2020). One of the most visible transformations has been the privatization of public spaces in cities. In this context, more and more “glocal” movements for transformation are appearing whose main claim is “the Right to the City” (Lefebvre, 1968 cited by Martínez, 2020a; Polanska and Weldon, 2020) as well as public spaces outside the market and the control of the capitalist state (Caffentzis and Federici, 2014 cited by Polanska and Weldon, 2020).

Urbanization has become a key tool for the development of capitalism, and the city plays a central role in capital accumulation (Novák and Kuřík, 2020; Yardımcı, 2020). Accelerated urbanization causes more and more people to move to cities (González et al., 2020; Novák and Kuřík, 2020); in turn, urban spaces are increasingly commodified, what has turned cities into neoliberal spaces where life is increasingly individualized and based on the free market (Novák and Kuřík, 2020).

The occupation of buildings or land is inherent to the practice of resisting commodification through challenging private property and institutional authority (Asara, 2019; Dadusc, 2019b; Starecheski, 2019; González et al., 2020; Polanska and Weldon, 2020). Occupation is thus an anti-capitalist expression of life, serving both as its own purpose (to meet the need for housing/shelter produced by the capitalist system) and as an instrument of resistance to neoliberalism (Raimondi, 2019; Soresina, 2020) that constructs a desirable future (Atabien and Tekdemir, 2020). Even when it is only an occupation to obtain housing, it is considered political by shaping an alternative to the imposition of the capitalist market (Caciagli, 2019).

#### Processes of political re-subjectivation through squatted spaces

The common life put into practice by the squatting movement, the communal learning, knowledge and experiences, all generates political subjects more disposed to collective existence (Lauri, 2019). While the neoliberal paradigm individualizes, squats work on the effects of depoliticization with ethical, social and political implications (Nicholls, 2016 cited by Caciagli, 2019), generating in turn a starting point for renewed political participation (D’Albergo and De Nardis, 2016 cited by Caciagli, 2019).

Cities have been identified as incubators of social movements. Increasingly, processes of politicization and depoliticization are studied in relation to urban contexts (De Moor, 2020), and it is established that there is an interplay between post-political forces, which depoliticize, and those that re-politicize through acts of opposition to the *status quo* (Novák, 2020).

Squatted spaces constitute urban community practices that, by forming autonomous communities of resistance to privatization and dispossession, generate new forms of governance that open up the possibility of radical political subjectivities to emerge (Dadusc, 2019b).

### Popular education and community outreach

Through the creation of self-organized spaces and communities as alternatives for living against capitalism, squatters generate commons for cities (Dadusc et al., 2019; Polanska and Weldon, 2020). For example, in these spaces it is particularly important the creation of explicit and implicit collectively accepted principles and rules that regulate behavior (Novák, 2020; Polanska and Weldon, 2020) and aim to unlearn what produces social exclusion (racism, sexism, and ableism). At the same time, they seek to accentuate behaviors of solidarity, self-determination and mutual help, creating spaces of freedom where everyone feels safe (Raimondi, 2019; Polanska and Weldon, 2020). This is how the creation of communities offers squatters an alternative to models of social reproduction (Polanska and Weldon, 2020).

The relationship with the surroundings and the neighborhood usually occurs when the space is already squatted and gradually becomes a space for external use such as the hosting of neighborhood support campaigns or cultural activities (Caciagli, 2019; Novák, 2020). This does not imply that relations between squatters and neighbors are easy, as the latter may react negatively to the squatters’ activities and put pressure on the authorities for eviction (Caciagli, 2019).

Beyond the services it offers, the main contributions that the squatting movement makes to the community are shared knowledge and learning (Ighe, 2020), as well as giving a voice to groups that are socially silenced by conditions of fear and dependence and that, through occupation, appropriate and inhabit urban, social, and political spaces (Dadusc, 2019a).

#### Urban commons

Occupation is understood within the urban *commons* as it provides and generates resources for the community (Dos Santos, 2020) that are highly valuable for anti-capitalist practices (Martínez, 2020b). *Commons* are characterized by property relations that reflect the collective decisions of the people who participate and use the resources (Rodrigo, 2010; Algarra, 2015; Sastre, 2018; Dos Santos, 2020). However, the available resources do not constitute *commons per se*, but become commons through collective organization (Dos Santos, 2020).

In the case of occupation, empty buildings are available resources, but this availability only exists outside the capitalist framework, only through occupation (Dos Santos, 2020). Occupation can be framed as a common when it is collective, cooperative, self-organized, based on mutual aid and non-exploitation, and is a survival practice for the working class (Martínez, 2020a).

### Squatters’ movement and migration

In 2015, the refugee crisis and the long migration summer formed a very powerful solidarity movement across Europe to address the hardship and rights violations of migrants, and to fight exclusion and racism (Martínez, 2016 cited by Maestri, 2019; Raimondi, 2019). In this context, movements such as the “We Are Here” in the Netherlands (Dadusc, 2019a), the “Syrian Solidarity House Initiative” and the “City Plaza” hotel in Athens (Martínez, 2020a; Zaman, 2020), Klinika in the Czechia (Novák, 2020) or Metropoliz in Italy (Martínez, 2020a) were created.

The increase of laws criminalizing migration is a technique of repression that extends illegality to all aspects of migrants’ lives, creating a hostile environment for them (Aas, 2011 cited by Dadusc, 2019a; Dadusc et al., 2019; Novák, 2020). Violence and coercion are not only produced by administrations (Dadusc, 2019b), but also by humanitarian borders (Walters, 2010 cited by Dadusc, 2019b) that treat migrants as an emergency, victimizing them and presenting them as vulnerable and in need from an apolitical approach (Dadusc, 2019b). In these cases, occupation represents a practice of resistance to criminalization and humanitarian borders, creating common spaces and solidarities against violence, segregation and the constraints of humanitarian security measures (Dadusc, 2019b; Martínez, 2020b; Karaliotas and Kapsali, 2021).

In addition, occupations with migrants or Roma together with activists have the function of making visible what has been excluded (Dikeç, 2012 cited by Maestri, 2019), creating spaces for silenced voices to be heard (City Plaza Refugee and Accommodation Space, 2017 cited by Raimondi, 2019). Although these shared occupations are based on a principle of equality, this does not prevent the appearance of internal conflicts or the reproduction of power relations (Dadusc et al., 2019; Karaliotas and Kapsali, 2021). Indeed, communities present tensions and contradictions in which they confront forms of racism and other internalized privileges, albeit with the possibility of learning from mistakes (Dadusc et al., 2019).

Inhabiting these spaces successfully overcomes isolation, dependency, the politics of fear and the silence of migrants and politicizes far beyond the coverage of accommodation needs in response to the austerity of neoliberalism (Dadusc, 2019a; Dadusc et al., 2019; Chiodelli et al., 2020; Zaman, 2020; Karaliotas and Kapsali, 2021). The occupation endows the illegal migration process with autonomy, constituting itself as a political movement that escapes institutions and delegitimize control and authority (Dadusc et al., 2019).

### Main social resistance toward the squatting movement

According to the literature analyzed, there are four main resistances that limit and threaten the existence and extension of the squatters’ movement.

Firstly, the stigmatization of the movement, which has been publicly delegitimized through its criminalization (Nowicki, 2020; Yardımcı, 2020), to which the media have contributed by portraying squatters as fanatics, criminal gangs, parasites, and invaders (Martínez, 2019). This has served political interests on the part of the state to continue urbanizing without encountering resistance (Kallin and Slater, 2014 citats per Yardımcı, 2020; Novák, 2020; Yardımcı, 2020).

Secondly, evictions, which lead to the loss of the squatted space, and force the squatting movement to develop strategies to face this constant threat (Caciagli, 2019). Very often, the eviction of a squatted space leads to the proliferation of other squats (Dadusc, 2019b). Therefore, instead of using direct forms of repression, institutions sometimes use more subtle governance strategies and offer negotiations to squatted spaces (Dadusc, 2019a; Lauri, 2019; De Moor, 2020). The institutionalization of many of the spaces as an alternative to eviction and because of these negotiations, ends up leading to control and surveillance, depoliticization of the movement and even police infiltration of social movements (Dadusc, 2019b; De Moor, 2020; Novák, 2020). Direct and indirect repression socially isolates squatted spaces, preventing them from accessing their necessary social bases (González et al., 2020). Yet, even in the face of eviction, activists have the capacity to challenge authority and politicize the debate about their own eviction (Novák, 2020).

Thirdly, internal conflicts that, although do not imply the failure of the project, represent resistance and alert of the need to maintain a critical view that avoids the reproduction of privileges and power structures (Atabien and Tekdemir, 2020; Karaliotas and Kapsali, 2021*).* Some squatted spaces present norms to minimize tensions between participants (Maestri, 2019; Novák, 2020; Polanska and Weldon, 2020). But breaking normative agreements can have serious implications, such as expulsion from the squatted space, so some people follow the rules not because they understand them as collectively generated processes, but out of fear of the consequence (Caciagli, 2019).

Finally, we find the precariousness of housing. The squatting movement offers a direct response to the need for housing, but often the occupied spaces are not suitable for living (Malik et al., 2020). The limitations of basic infrastructure make conditions unfavorable for continued occupation (Malik et al., 2020).

## Discussion

The main thematic focuses addressed by the scientific literature on the squatting movement refer to its conceptualization and classification, its counter-hegemonic role and resistance to neoliberalism and capitalism, its links with the community and with the anti-racist and migrant movements, and, finally, its limitations and the resistance it can generate.

Since its emergence between the 1960s and 1970s, the squatting movement has materialized two distinct lines of social transformation. On the one hand, the occupation is finalist insofar as it creates alternatives to cover basic housing needs regardless of the ethnicity, gender identity, age, or personal or legal situation of all the people who participate. In this way, the movement contributes to changing the material reality of the people who squat. On the other hand, the squatting movement is also a tool for counter-hegemonic transformation. Through occupation, processes of learning and formation of political subjects are generated that allow squatters and all those who come into contact with squatted spaces to rethink the power structures and hegemonic social roles that are characteristic of the capitalist system. In this sense, it contributes to social transformation through critique and the construction of alternative spaces and self-managed communities.

The interrelation between the squatters’ movement and other social movements, with which it weaves networks of solidarity and support, contributes to social transformation through the collective construction of knowledge and the generation of open spaces for political participation. In particular, the scientific literature highlights the relationship with the migration and anti-racist movements. In a context of the promotion of economically and politically exclusionary policies, occupation represents one of the main alternatives to institutional humanitarian aid, which often victimizes and violates the autonomy of migrants.

The practices of self-management, training and linking with the environment allow us to understand squatted spaces as places where popular education initiatives can be developed in which the objectives and the ways of achieving them are decided collectively; where the participation of the whole community is stimulated; where all voices have the same opportunities to express themselves and are considered by the group without prejudice.

Even so, we cannot ignore the fact that the relationship between activists from the squatting movement and the migrant and anti-racist movements is not always easy. Cultural and ideological differences, as well as the diversity of motivations for squatting, often lead to the emergence of internal conflicts, especially between activists and migrants or refugees, which can deteriorate coexistence in the squatting space and make internalized power structures visible, especially on the part of the activists. These same structures (racist, sexist, and classist) can also affect squatting collectives internally.

Thus, internal conflicts constitute one of the main limitations for the squatting movement. While some authors consider that conflict is part of the process of coexistence and that it provides opportunities for learning and questioning social structures, for other authors it can become the cause of the termination of the squatted space project, having a negative impact on the community (Van der Steen et al., 2020). Reinforcing the weight of popular education in the management of these conflicts can facilitate reflection on the privilege of some activists in relation to others and provide the movement with tools to overcome it (Llobet, 2004).

Repression and stigma are also important constraints for the movement. Evictions are the most direct form of repression against the movement, and its main threat, as they deprive it of the space in which to develop its political activity. However, there are other -indirect- forms of repression, such as the hypervigilance of squatted spaces or the attempts to depoliticize projects. This depoliticization can materialize through an institutional appropriation of the contents of the Right to the City (Mayer, 2012) and of the spaces and practices of previously squatted centers that are now managed by the administration. Stigma can also be understood as a form of indirect repression that can make it difficult for the movement to gain support.

As for the political orientation of the movement, all the studies included in this review that talk about squatting from a political perspective do so about squatted and politicized left-wing spaces. While these make up the vast majority of the movement, there are also political spaces that defend other positions. Even so, Martínez (2020b) establishes that when the occupation is carried out by individuals with the intention of enriching themselves, or by extreme right-wing movements, it is not included within the squatting movement. Although these practices can be understood as counter-hegemonic if they subvert basic principles of the established social order, it is understood that they cannot be considered part of the movement due to the absence of a social emancipation project that characterizes it.

Finally, it is necessary to mention that the studies included in the review are mainly focused on Europe. This indicates that the scientific literature in the languages included in this article is limited to the study of the European movement, which is, in turn, a limitation in our own review.

## Conclusion

Recovering the guiding questions posed in the introduction to this article: What is the squatting movement?, What are its main dynamics of resistance?, How does its process of political subjectivation take place?, and What are its main mechanisms of action and self-management? We can conclude that there is a large amount of scientific literature that provides evidence of cases in which the squatting movement has or has had an impact on the social transformation of the context and the material reality of the neighborhoods.

We can conclude, by consensus of the majority of authors, that all collective occupation constitutes a political process and creates alternatives for housing, socialization and culture in the face of the commodification of public space and housing speculation by the administrations of the capitalist and neoliberal system.

The main contributions of the squatting movement, according to the literature, are firstly, the direct response to the social needs detected: whether covering basic needs such as housing or generating non-commercialized spaces for socialization and directly challenging private property, which is fundamental for profit and the accumulation of capital. In other words, one of the main contributions is the creation of *commons.*

Secondly, the opening of the squatted space generates processes of political subjectivation that allow for the questioning of established power structures and social roles. This allows for the deconstruction of internalized ideas at a personal and relational level because of the hegemonic structures of the capitalist and neoliberal system. The squatting movement promotes values of solidarity, cooperation, and anti-capitalism, which have as their ultimate goal the politicization of the working class for social transformation.

Finally, the occupation creates safe self-managed spaces for dissident people and identities, seeks to give a voice and to listen through daily practice, self-governance and political and protest action to those voices that are never heard, thus transforming the reality of these people.

Based on the literature, we can establish that another contribution of the squatting movement to social transformation is generated through its interaction with other social movements, serving as a tool for them. One of the interrelationships on which the scientific literature has focused the most is the one between the squatting movement and the migrant-anti-racist movement. Occupation becomes a response to migratory and discriminatory policies, an alternative for asylum, community building and socialization for migrants and refugees whose rights are violated, who are criminalized and hyper-policed by states, and whose political and social participation is limited by humanitarian borders.

Based on the evidence and research carried out so far, we believe it would be useful to expand knowledge about the squatter movement and social transformation in territorial contexts outside Europe in the future. This would also broaden the available knowledge on the movement on a global level and allow comparisons to be made between the conditions and contributions of the movement in different cultural, economic, political, and social contexts.

## Data availability statement

The original contributions presented in this study are included in the article/supplementary material, further inquiries can be directed to the corresponding author.

## Author contributions

JB-Q, PR-V, and JJ contributed to the conception and design of the study, selected the articles, and read and categorized the data. JB-Q and PR-V organized the database. JB-Q performed the statistical analysis and wrote the first draft of the manuscript. PR-V and JJ wrote sections of the manuscript, and reviewed and organized the references. All authors contributed to manuscript revision, read, and approved the submitted version.
