# Corrigendum: Counter hegemony, popular education, and resistances: A systematic literature review on the squatters' movement

**DOI:** 10.3389/fpsyg.2022.1097795

**Published:** 2022-11-29

**Authors:** Julia Ballesteros-Quilez, Pablo Rivera-Vargas, Judith Jacovkis

**Affiliations:** ^1^Faculty of Psychology, University of Barcelona, Barcelona, Spain; ^2^Department of Teaching and Learning and Educational Organization, University of Barcelona, Barcelona, Spain; ^3^Facultad de Educación y Ciencias Sociales, Universidad Andrés Bello, Santiago, Chile

**Keywords:** squatting movement, squatters, anti-capitalism, social movements, neoliberalism, self-management, insurgency, social transformation

In the published article, there was an error in the **Funding** statement. The **Funding** statement for the article was displayed as “The University of Barcelona provided the Open Access funding (UB-AE-2022).” The correct **Funding** statement appears below.

## Funding

This research has received funding from the UB-AE-AS017654 call, and the support of the UB funding for Open access publishing.

The authors apologize for this error and state that this does not change the scientific conclusions of the article in any way. The original article has been updated.

## Publisher's note

All claims expressed in this article are solely those of the authors and do not necessarily represent those of their affiliated organizations, or those of the publisher, the editors and the reviewers. Any product that may be evaluated in this article, or claim that may be made by its manufacturer, is not guaranteed or endorsed by the publisher.

